# Supplementary material for: Differential Associations of Intakes of Whole Grains and Coarse Grains with Risks of Cardiometabolic Factors among Adults in China
Source: Nutrients. 2022 May 18;14(10):2109. doi: 10.3390/nu14102109 (PMC9145902; doi:10.3390/nu14102109)
Supplement: Supplementary file 1 [file nutrients-14-02109-s001.zip › nutrients-1693884-supplementary.pdf]

Table S1. Baseline characteristics of participants across quartiles of coarse grain intake, CHNS 2011

	Total(n=4706)	Nonconsumer(n=3357)	T1(n=517)	T2(n=402)	T3(n=430)	*p*-value
Coarse grains (g/day)	—	—	20.00(13.33,33.33)	50.00(46.67,63.33)	116.67(90.00,173.07)	<0.001
Gender, n(%)[b]						0.362
Men	2108(44.79)	1530(45.58)	217(41.97)	173(43.03)	188(43.72)	
Women	2598(55.21)	1827(54.42)	300(58.03)	229(56.97)	242(56.28)	
Age, n(%)						0.011
18-44 years	1648(35.02)	1194(35.57)	194(37.52)	124(30.85)	136(31.63)	
45-64 years	2407(51.15)	1707(50.85)	267(51.64)	202(50.25)	231(53.72)	
≥65 years	651(13.83)	456(13.58)	56(10.83)	76(18.91)	63(14.65)	
Income level, n(%)						0.342
Low	1607(34.15)	1142(34.02)	180(34.82)	143(35.57)	142(33.02)	
Medium	1607(34.15)	1173(34.94)	177(34.24)	120(29.85)	137(31.86)	
High	1492(31.70)	1042(31.04)	160(30.95)	139(34.58)	151(35.12)	
Education, n(%)						0.002
< Primary school	1808(38.42)	1317(39.23)	155(29.98)	160(39.80)	176(40.93)	
Primary school	1568(33.32)	1093(32.56)	192(37.14)	141(35.07)	142(33.02)	
> Primary school	1330(28.26)	947(28.21)	170(32.88)	101(25.12)	112(26.05)	
Urbanicity index, n(%)						<0.001
Low	1795(38.14)	1222(36.40)	167(32.30)	178(44.28)	228(53.02)	
Medium	1595(33.89)	1199(35.72)	173(33.46)	118(29.35)	105(24.42)	
High	1316(27.96)	936(27.88)	177(34.24)	106(26.37)	97(22.56)	
Smoking, n(%)	1400(29.75)	1025(30.53)	125(24.18)	124(30.85)	126(29.30)	0.030
Drinking, n(%)	1616(34.34)	1152(34.32)	181(35.01)	135(33.58)	148(34.42)	0.976
Physical activity (MET hours/week)3	221.06±14.05	220.83±3.26	210.72±8.32	222.87±9.43	233.60±9.12	0.324
Dietary intake						

Total energy (kcal/day)	2119.00±53.47	2102.57±12.42	2031.29±31.65	2170.49±35.90	2304.62±34.71	<0.001
Total grains (g/day)	395.89±15.65	380.44±3.02	361.91±7.71	433.08±8.74	522.64±8.48	<0.001
Rice and rice products (g/day)	247.14±15.12	281.12±2.92	173.28±7.45	173.14±8.45	139.82±8.19	<0.001
Wheat and wheat products (g/day)	127.64±12.07	99.26±2.33	166.77±5.95	206.92±6.74	228.05±6.54	<0.001
Tuber (g/day)	30.82±5.31	28.42±1.03	28.38±2.62	34.53±2.97	49.08±2.88	<0.001
Red meat (g/day)	95.43±6.77	104.99±1.31	85.22±3.34	67.59±3.79	59.16±3.67	<0.001
Poultry (g/day)	19.33±3.55	21.46±0.69	16.67±1.75	14.13±1.99	10.76±1.93	<0.001
Fish (g/day)	33.12±4.81	37.01±0.93	21.63±2.37	23.46±2.69	25.61±2.61	<0.001
Vegetables and fruits (g/day)	394.15±18.15	400.45±3.51	374.98±8.95	367.51±10.14	392.96±9.83	0.002
Cooking oil (g/day)	42.90±2.73	43.73±0.53	44.62±1.35	41.60±1.53	35.50±1.48	<0.001
Sodium (mg/day)	5542.00±821.34	5432.28±158.73	5483.95±404.81	6805.72±458.97	5287.00±444.90	0.038

[a] T = tertile; CMFs= cardiometabolic factors; MET=metabolic equivalent.

[b] Data are number of participants (%).

[c] Mean ± standard error (all such values). Adjusted by age for total energy intake, Physical activity and adjusted by age and total energy intake for other food groups .

[d] Chi-square tests for categorical variables and general linear models for continuous variables to test statistical significance of differences among groups .

Table S2. Prevalence of CMFs in 2015 among Chinese adults across baseline levels of coarse grains intake

	Total(n=4706)	Nonconsumer(n=3357)	Consumers			p-trend
			T1(n=517)	T2(n=402)	T3(n=430)	
CMFs cluster, n(%)	3629(78.43)	913(78.98)	935(80.40)	902(77.96)	879(76.37)	0.258
Abdominal obesity, n(%)	2307(49.86)	619(53.55)	607(52.19)	572(49.44)	509(44.22)	<0.001
Overweight, n(%)	2167(46.83)	530(45.85)	539(46.35)	563(48.66)	535(46.48)	0.040
Elevated BP, n(%)	2226(48.11)	502(43.43)	550(47.29)	612(52.90)	562(48.83)	0.021
Elevated FBG, n(%)	1385(29.93)	326(28.20)	358(30.78)	335(28.95)	366(31.80)	0.284
Insulin resistance, n(%)	653(14.11)	188(16.26)	169(14.53)	154(13.31)	142(12.34)	0.149
LR factors, n(%)	2782(60.13)	745(64.45)	711(61.13)	684(59.12)	642(55.78)	0.778
Elevated TG, n(%)	1112(24.03)	261(22.58)	315(27.09)	278(24.03)	258(22.42)	<0.001
Reduced HDL-C, n(%)	1682(36.35)	466(40.31)	459(39.47)	407(35.18)	350(30.41)	0.623
Elevated LDL-C, n(%)	1644(35.53)	449(38.84)	407(35.00)	391(33.79)	397(34.49)	0.666

[a] T = tertile; CMFs= cardiometabolic factors; BP= blood pressure; FBG= fasting blood glucose; LR factors= lipid-related factors; TG=triglycerides; HDL-C=high-density lipoprotein cholesterol; LDL-C=low-density lipoprotein cholesterol; HOMA-IR=[fasting insulin (mU/mL)*fasting blood glucose (mmol/L)]/22.5.

[b] Significance for the prevalence of each CMFs were determined by using Chi-square test ($^{*}p<0.05$), and by using Cochran and Mantel-Haenszel to test trend across tertils of whole grain intake.
